# Pregnancy and Lifestyle: Short- and Long-Term Effects on Mother's and Her Children's Health

**DOI:** 10.1155/2013/537526

**Published:** 2013-05-28

**Authors:** Riitta Luoto, Michelle F. Mottola, Leena Hilakivi-Clarke

**Affiliations:** ^1^UKK Institute for Health Promotion Research, 33501 Tampere, Finland; ^2^Department of Children and Families, National Health and Welfare Institute, 00271 Helsinki, Finland; ^3^R. Samuel McLaughlin Foundation-Exercise and Pregnancy Lab, 3-M Centre, The University of Western Ontario, London, ON, Canada N6A 3K7; ^4^Georgetown University, Research Building, Room E407, 3970 Reservoir Road, NW, Washington, DC 20057, USA

Pregnancy and the foetal environment can have a profound influence on many chronic diseases, such as diabetes obesity, breast cancer, and cardiovascular diseases in both the mother and her offspring. Much of the influence of the intrauterine milieu is transmitted to the next generation through epigenetic mechanisms. However, the specific pathways affected through these mechanisms by lifestyle during pregnancy are largely unknown and deserve further study.

The special issue presents the latest findings and reviews from all over the world concerning pregnancy and lifestyle, especially nutrition, physical activity, and weight gain during pregnancy. The special issue includes three reviews, two of which cover the role of maternal physical activity, under- and overnutrition, and intake of specific nutrients during pregnancy on long-term risk of chronic diseases among mother and her offspring. The third review article concentrates on induction and prevention of cardiovascular birth defects. The first month of gestation is the most important for development of cardiovascular birth defects that may be preventable by folate acid supplementation.

The original articles in this special issue cover topics from lifestyles (physical activity and nutrition) to chronic diseases and predisease states, such as metabolic syndrome, during pregnancy. The only animal study included in the special issue describes findings showing how maternal hyperglycaemia is related to foetal pancreatic function, resulting in inadequate insulin secretion postnatally. In a human study included in this issue, it was reported that women at high risk of gestational diabetes were at increased risk of developing metabolic syndrome, which includes central obesity, dyslipidemia, and blood glucose abnormalities, already one year postpartum.

Lifestyle modifications during or after pregnancy have been suggested to be valuable in chronic disease prevention. Further, these modifications may have to take place before pregnancy. Prenatal education programs have been studied in Ontario. Canadian researchers recommend that prenatal education programs should include information on appropriate gestational weight gain targets that can be achieved by lowering energy intakes and being physically active. According to the Norwegian HUNT study, women with high prepregnancy body mass index have an increased risk of having macrosomic offspring, if their physical activity was low as well.

According to researchers from Iowa, in the USA, existing physical activity guidelines during pregnancy vary, and further research is warranted to identify health (mother and offspring) enhancing physical activity and relevant pregnancy outcomes. This is important, since pregnant women in a Norwegian hospital-based study had high motivational readiness or intention to increase their physical activity level. Pregnancy can therefore be considered a “window of opportunity” for long-term physical activity habits. New information on pregnant women's physical activity was reported by using kinematic analysis in Portugal. Pregnant women need to maintain greater stability of body and increase the efficiency of physical activity.

Finally, pregnant women are exposed to other lifestyle-related threats besides poor nutrition and lack of physical activity, which should be monitored as well. A Brazilian birth cohort study reported that half of pregnant women used at least one medicine with an unknown fetal risk. It is well reported that smoking during pregnancy is harmful for a fetus, but a Danish National Birth Cohort study was unable to establish a connection between smoking during pregnancy and offspring's intelligence at the age of 5. Later consequences of lifestyle during pregnancy may be difficult to separate from genetic profiles and other determinants, which should be investigated in future studies.

*Riitta Luoto* *Riitta Luoto* *Michelle F. Mottola* *Michelle F. Mottola* *Leena Hilakivi-Clarke* *Leena Hilakivi-Clarke*

